# Salvianolic Acid Modulates Physiological Responses and Stress-Related Genes That Affect Osmotic Stress Tolerance in *Glycine max* and *Zea mays*

**DOI:** 10.3389/fpls.2022.904037

**Published:** 2022-06-15

**Authors:** Elham Ahmed Kazerooni, Abdullah Mohammed Al-Sadi, Umer Rashid, Il-Doo Kim, Sang-Mo Kang, In-Jung Lee

**Affiliations:** ^1^Department of Applied Biosciences, Kyungpook National University, Daegu, South Korea; ^2^Department of Plant Sciences, College of Agricultural and Marine Sciences, Sultan Qaboos University, Muscat, Oman; ^3^Institute of Nanoscience and Nanotechnology (ION2), Universiti Putra Malaysia, Serdang, Malaysia

**Keywords:** hydrogen peroxide, lipid metabolism, fatty acid, amino acid, antioxidant enzymes, sugar, protein

## Abstract

Drought is a serious threat worldwide to soybean and maize production. This study was conducted to discern the impact of salvianolic acid treatment on osmotic-stressed soybean (*Glycine max* L.) and maize (*Zea mays* L.) seedlings from the perspective of physiochemical and molecular reactions. Examination of varied salvianolic acid concentrations (0, 0.1, 1, 5, 10, and 25 μM) on soybean and maize seedling growth confirmed that the 0.1 and 1 μM concentrations, respectively, showed an improvement in agronomic traits. Likewise, the investigation ascertained how salvianolic acid application could retrieve osmotic-stressed plants. Soybean and maize seedlings were irrigated with water or 25% PEG for 8 days. The results indicated that salvianolic acid application promoted the survival of the 39-day-old osmotic-stressed soybean and maize plants. The salvianolic acid-treated plants retained high photosynthetic pigments, protein, amino acid, fatty acid, sugar, and antioxidant contents, and demonstrated low hydrogen peroxide and lipid contents under osmotic stress conditions. Gene transcription pattern certified that salvianolic acid application led to an increased expression of *GmGOGAT, GmUBC2, ZmpsbA, ZmNAGK, ZmVPP1*, and *ZmSCE1d* genes, and a diminished expression of *GmMIPS2, GmSOG1, GmACS, GmCKX, ZmPIS*, and *ZmNAC48* genes. Together, our results indicate the utility of salvianolic acid to enhance the osmotic endurance of soybean and maize plants.

## Introduction

Climate change has increased the prevalence of various abiotic stress conditions around the world (Pryor et al., 2014; Ogata et al., 2017). A vast range of stressful environmental stimuli, such as salinity, ultraviolet radiation, heat, flooding, drought, and heavy metals, pose a serious threat to plants (He et al., 2018; Keep et al., 2021; Liu et al., 2021, 2022; Sheteiwy et al., 2021c; Simioniuc et al., 2021). These stress factors are the major constraints for crop survival, accounting for a dramatic reduction in crop yield globally (Lamers et al., 2020; Sheteiwy et al., 2021a). Drought is among the most prevalent abiotic stress condition that adversely influences plant growth, quality, and yield (Thirumalaikumar et al., 2018; Du et al., 2020b). Different factors, including water deficiency, high temperature, and low humidity, can be induced by drought conditions (Bartels et al., 2004; Abdoulaye et al., 2019). Drought stress can intrude various physiological processes, namely, plant photosynthesis, oxidative stress, enzyme activity, nucleic acids, proteins, membrane integrity, and cell metabolism (Valliyodan and Nguyen, 2006; Sheteiwy et al., 2021c), which could subsequently result in the prevention of plant growth.

*Salvia miltiorrhiza* Bunge (Lamiaceae), known as red sage, has been clinically applied in traditional Chinese medicine for more than 2,000 years. In recent years, it has been extensively approved as a health product in western countries (Ma et al., 2019). It is consumed as a drug for cardiovascular disorders, dysmenorrhea, angina pectoris, cancer, thrombosis, hepatitis, hepatocirrhosis, and neurasthenic insomnia (Li, 1998; Wang et al., 2017). Phytochemical studies have demonstrated that *S. miltiorrhiza* is composed of large amounts of compounds with strong anti-oxidative activity, including flavonoids, polyphenols, triterpenoids, lipophilic diterpenoids, and phenolic compounds (such as salvianolic acids) (Lu and Foo, 2002; Li et al., 2009). Salvianolic acids are the most water-soluble compounds in *S. miltiorrhiza*, and among them, salvianolic acid A and salvianolic acid constitute the most abundant compounds (Ma et al., 2019). It has been reported that salvianolic acids exhibited antioxidative properties and free radical scavenging activities in *in vitro* and *in vivo* conditions, and showed protective effects on cells exposed to detrimental agents (Zhao et al., 2008; Zhang et al., 2014).

Soybean (*Glycine max* L.) and maize (*Zea mays* L.) are the most substantial feed crops cultivated around the world. These crops contain beneficial metabolites and nutrients, which prevent cancer, kidney diseases, obesity, and diabetes (Messina, 2016; Mao et al., 2021; Poku et al., 2021). Soybean is considered as an ample source of oil and protein for humans. In addition to their consumption, soybean and maize are considered a future source of fuel and alternative for plastics, respectively (Candeia et al., 2007; Song et al., 2011; Wang et al., 2016). Despite these benefits, the growth and productivity of soybean and maize are substantially interfered by various abiotic stress factors (Deshmukh et al., 2014; Li et al., 2019; Mao et al., 2021). Amid the detrimental environmental factors usually faced by soybean and maize, drought is believed to be the harshest, since it influences all phases of plant development and subsequently reduces the final yield (Le et al., 2012; Yang et al., 2018). Thus, research on enhancing the growth and endurance of soybean and maize plants under drought stress is important to diminish the effect of water deficit and improve crop yield (Sheteiwy et al., 2021a,b).

Evaluations on the incorporation of climate alteration and crop yield models have anticipated greater loss in the production of major crops, including rice, soybean, wheat, and maize, which may have severe consequences for food safety (Waqas et al., 2019). We envisaged that salvianolic acid could promote osmotic stress survival in soybean and maize plants under destructive environmental situations. This work was conducted to examine the impact of the salvianolic acid application on attenuating osmotic stress in soybean and maize plants and determine its effect on plant development and production. We attempted to specify the appropriate salvianolic acid concentration that was efficient toward osmotic-stressed plants. In this study, physiochemical and molecular analyses were employed to perceive the mechanisms of salvianolic acid in soybean and maize plants under osmotic stress conditions. In particular, we show how exogenous salvianolic acid application influences the sugar content, amino acid content, fatty acid content, and transcription patterns of various genes. Our work presents a convincing demonstration of the positive effects of salvianolic acid on ameliorating the osmotic stress tolerance in soybean and maize plants.

## Materials and Methods

### Determination of Proper Concentrations of Polyethylene Glycol

Soybean and maize seeds were rendered by the Agricultural Research and Extension Services (Gyeongsangbuk-do, South Korea) and Asia Seed Ltd. (Seoul, South Korea), respectively. Similar-sized seeds were selected and disinfected in 70% ethanol and 2.5% sodium hypochlorite and evaluated for vitality (Ke et al., 2018; Silva et al., 2020). Seeds were transferred onto pot trays (28 × 54 cm) filled with horticultural soil (Shinsung Mineral Co., Ltd., Chungcheongbuk-do, Korea), grown in a climate chamber (16/8 h day/night), and irrigated daily. The flow rate, humidity, and temperature in the chamber were maintained at 250 μmol m^−2^ s^−1^, 65%, and 26 ± 2°C, respectively. Following emergence, one plant per pot (10 × 10 cm) received assorted treatments. Maize and soybean seedlings were either irrigated with distilled water (50 ml/pot), as control, or with polyethylene glycol 6,000 (PEG-6000, Merck-Schuchardt, Hohenbrunn, Germany) at −0.15, −0.3, −0.49, and −0.73 MPa concentrations (50 ml/pot). We used PEG-6000 to simulate drought stress conditions (PEG-generated osmotic stress). Each group comprised six replicates. After the completion of each treatment period (8DAT), plants were assayed for various agronomic characteristics. Finally, 25% polyethylene glycol 6,000 (PEG-6000; −0.73 MPa) was designated to be the right concentration for use in further experiments.

### Determination of the Suitable Salvianolic Acid Concentration

Average-sized seeds were surface-sterilized (Ke et al., 2018; Silva et al., 2020) and grown in a climate chamber as described earlier. Next, maize and soybean seedlings were partitioned into two groups: (i) control, which received 50 ml/plant of distilled water, and (ii) osmotic stress treatment, irrigated with 25% PEG (50 ml/plant) and exposed to these treatments for 8 days. Later, the osmotic-stressed seedlings were treated with 50 ml per plant of 0, 0.1, 1, 5, 10, and 25 μM salvianolic acid (Sigma-Aldrich, St. Louis, Missouri, USA) daily for 8 days. The salvianolic acid solution (25 μM, stock) was prepared by dissolving the solute in water, and then the stock solution was diluted in distilled water to obtain different concentrations. Each treatment contained six replicates. All the plant growth characteristics were measured after 8 days (8DAT). Subsequently, 0.1 and 1 μM salvianolic acid (SAL) concentrations were identified to be the appropriate concentrations for further experiments.

### Physiochemical and Molecular Effects of Salvianolic Acid on Osmotic-Stressed Soybean and Maize Seedlings

#### Growth Condition and Treatments

The soybean and maize seedlings were maintained in a greenhouse at 43% relative humidity and 25/23°C day/night temperature. Three weeks after emergence, plantlets with similar maturity were used in this study. Plantlets (one seedling/pot, irrigated with 50 ml/pot) were irrigated with distilled water, 0.1 μM SAL, 1 μM SAL, or 25% PEG, according to Kazerooni et al. (2021) (Table 1). Each treatment consisted of six replicates. The maize and soybean seedlings were consistently irrigated with SAL, and leaves were collected 8 days after treatment. The maize and soybean leaves were promptly used or deep-frozen in liquid nitrogen before storage at −80°C.

**Table 1:** Experimental work plan.

**Symbol**	**Treatment**
**Soybean**	
Cont	irrigated with sterile distilled water
SAL	irrigated with 0.1 μM SAL
PEG	irrigated with 25% PEG
PEG+SAL	irrigated with 25% PEG + 0.1 μM SAL
**Maize**	
Cont	irrigated with sterile distilled water
SAL	irrigated with 1 μM SAL
PEG	irrigated with 25% PEG
PEG+SAL	irrigated with 25% PEG + 1 μM SAL

#### Measurement of Physiological Characteristics and Chlorophyll Index

Diverse agronomic traits were measured to determine the impact of one-by-one treatment on the soybean and maize seedlings. These traits were recorded 8 days after treatment. A digital Vernier caliper and a ruler were used to measure the stem diameter and leaf area (leaf length/width). Plant height and root length were assessed with a tape meter. In primary osmotic stress and SAL screening test, a SPAD meter (SPAD-502, Konica Minolta, Tokyo, Japan) was employed to determine the chlorophyll concentration in leaves. The plants and roots were oven-dried at 60°C for 48 h to assess their dry weights (Valentovic et al., 2006).

#### Changes in Chlorophyll, Carotenoid, and Amino Acid Contents

The contents of chlorophyll a (Chl a), chlorophyll b (Chl b), total chlorophyll (Total Chl), and carotenoid were determined according to Hosseini et al. (2017). Freshly harvested leaves (0.5 g) were immersed and homogenized in 80% acetone solution (20 ml). The absorbance of the extract was then recorded at the selected wavelength using a spectrophotometer (Thermo Fisher Scientific, Waltham, MA, USA).

Waqas et al. (2015) proposed a method for determining the amino acid content. Powdered freeze-dried leaves (50 mg) were hydrolyzed with 1 ml of hydrochloric acid (6 N HCl, 24 h, 110°C), followed by evaporation and condensation under vacuum (80°C, 24 h). Then, hydrochloric acid (1 ml of 0.02 N HCl) was added to dissolve the condensed remnant. The extract was filtered (0.45-μm membrane) before loading into Amino Acid Analyzer (Hitachi High-Technologies Corporation, Tokyo, Japan).

#### Hydrogen Peroxide, Lipid Peroxidation, and Fatty Acid Analysis

The concentration of H_2_O_2_ was evaluated based on a modified method (Velikova et al., 2000) originally described by Kazerooni et al. (2021). The powdered leaf tissue (0.3 g) was homogenized in an ice bath with 0.1% trichloroacetic acid (TCA; 5 ml). The absorbance of the mixture was recorded at 390 nm using a spectrophotometer.

López-Serrano et al. (2019) method was applied to evaluate the lipid content in soybean and maize leaves. The mixture comprising 0.1% TCA (0.5 ml) extract was added to 0.5% TBA (1 ml; prepared in 20% TCA). The reaction was commenced by incubating the mixture at 95°C for 30 min and terminated by placing the mixture in an ice bath for 10 min. Then the mixture was immediately centrifuged at 12,000 rpm for 5 min. Lipid content was measured at wavelengths of 532 and 600 nm (Thermo Fisher Scientific, Waltham, MA, USA).

We used a previously published method (Poirier et al., 1999) to determine the fatty acid content in soybean and maize leaves. Gas chromatography–mass spectrometry analysis was conducted on an Agilent Model 7890A series (Agilent, Dover, DE, USA).

#### Protein and Sugar Content Quantification

The concentration of protein was assessed using the Brilliant Blue G-250 reagent with bovine serum albumin following Bradford's method (Bradford, 1976).

The soluble sugar content of leaves was quantified according to a former report (Du et al., 2020b). A ground sample (0.1 g) was extracted with 80% (v/v) ethanol (at 80°C for 30 min) and then centrifuged at 10,000 rpm for 10 min. The obtained remnant was extracted twice utilizing 80% ethanol. The collected supernatants were mixed, and 80% ethanol was added to reach a final volume of 5 ml. Then, the soluble sugar content was quantified at a wavelength of 620 nm using a spectrophotometer.

#### Measurement of Antioxidant Activities

The polyphenol oxidase (PPO) and peroxidase (POD) activities were assayed following the method described by Putter (1974). Catalase (CAT) and superoxide dismutase (SOD) activities were inspected according to de Azevedo Neto et al. (2005). Flavonoids, DPPH radical scavenging performance, and total polyphenols were assessed according to Zheng and Wang (2001), Barka et al. (2006), Wang et al. (2009), and Kazerooni et al. (2021). The absorbance of the reaction mixture was characterized at preferred wavelengths using a spectrophotometer.

#### Estimation of Nutrient Content in Soybean and Maize Plants

Collected samples were freeze-dried and powdered to unravel the nutrient content of soybean and maize plants. The nutrient (potassium, K; phosphorus, P; and calcium, Ca) concentration in plants was determined using inductively coupled plasma mass spectrometry (Optima 7900DV, Perkin-Elmer, Akron, OH, USA). Treatments without osmotic stress or salvianolic acid were used to inspect the initial concentration of the selected elements.

#### RNA Extraction and Expression Analysis

Total RNA from leaves of soybean and maize at 8DAT was isolated using Trizol reagent (Thermo Fisher Scientific, Waltham, Massachusetts, USA). cDNAs were generated from total RNA (1 μg) using BioFACT RT-Kit (BIOFACT, Daejeon, Korea) according to the manufacturer's conventional instructions. qRT-PCR was performed with an Illumina Ecosystem (Illumina, San Diego, CA, USA) to determine the relative transcript levels of the selected genes. Primer sequences used for qRT-PCR are given in Supplementary Tables S1, S2.

### Statistical Analysis

The SAS statistical software (version 9.4, SAS Institute, Cary, NC, USA) was utilized to evaluate the data through analysis of variance (ANOVA). Substantial contradictions between treatments were clarified using Tukey's test at *p* < 0.05. All data are shown as six biological replicates. Graphs are drawn using Origin Pro (version 9.85, Origin Lab Corporation, Northampton, MA, USA).

## Results

### The Growth Changes of Soybean and Maize Seedlings Under Varied Polyethylene Glycol Concentrations

Diverse plant growth characteristics were recorded in soybean and maize plants exposed to different concentrations of PEG (−0.15, −0.3, −0.49, and −0.73 MPa). In general, they exhibited a decline in plant growth parameters (Supplementary Figures S1, S2 and Supplementary Tables S3, S4). Soybean and maize plants showed no evident changes when treated with the lowest concentration of PEG (−0.15 MPa) when compared to the control plants. However, treatment with maximum PEG concentration (-0.73 MPa) depicted considerable mitigation in the growth attributes of soybean and maize plants. For instance, attenuated plant height (58.50%), root length (50%), stem diameter (57.89%), leaf length (59.59%), leaf width (68.33%), chlorophyll content (51.22%), plant fresh weight (88.49%), plant dry weight (89.47%), root fresh weight (93.38%), root dry weight (100%), and leaf number (52.60%) (*p* < 0.05) were recorded in PEG-treated maize plants (-0.73 MPa) in contrast to control plants (Supplementary Figure S2 and Supplementary Table S4). These data showed that PEG (−0.73 MPa) decreased the plant growth of maize and soybean noticeably.

### Agronomic Traits of Stressed Soybean and Maize Seedlings Under Diverse Salvianolic Acid Concentrations

Mitigation in the varied plant growth parameters during osmotic stress is shown in Supplementary Table S5. On the other hand, when osmotic-stressed plants were treated with different concentrations of SAL (0, 0.1, 1, 5, 10, and 25 μM), they significantly displayed alleviation of osmotic stress (Supplementary Figures S3, S4 and Supplementary Table S5). At 8DAT, osmotic-stressed maize plants treated with a minimum (0.1 μM) or a maximum concentration of SAL (25 μM) showed no significant fluctuation when compared to stressed soybean plants (Supplementary Figure S4 and Supplementary Table S5). However, osmotic-stressed maize plants irrigated with SAL (1 μM concentration) began to display enhanced plant growth characteristics. By 8DAT, elevated plant height (69.23%), root length (53.57%), stem diameter (54.16%), leaf length (75%), leaf width (60.40%), chlorophyll content (36.49%), plant fresh weight (89.63%), plant dry weight (96.35%), root fresh weight (80.72%), root dry weight (75%), and leaf number (42.85%) (*p* < 0.05**)** were observed in SAL-treated stressed maize plants in contrast to osmotic-stressed plants alone (Supplementary Figure S4 and Supplementary Table S5). In addition, stressed soybean plants irrigated with a minimum concentration of SAL (0.1 μM) showed marked improvement in their growth parameters compared to osmotic-stressed plants alone (Supplemantary Figure S3 and Supplementary Table S5). These outcomes suggested that the osmotic stress was repressed in soybean and maize plants treated with 0.1 and 1 μM SAL, respectively.

### Effect of Salvianolic Acid on Stressed Soybean and Maize Seedlings

#### Salvianolic Acid Improves Plant Growth Attributes Under Osmotic Stress

**Figure 1:**
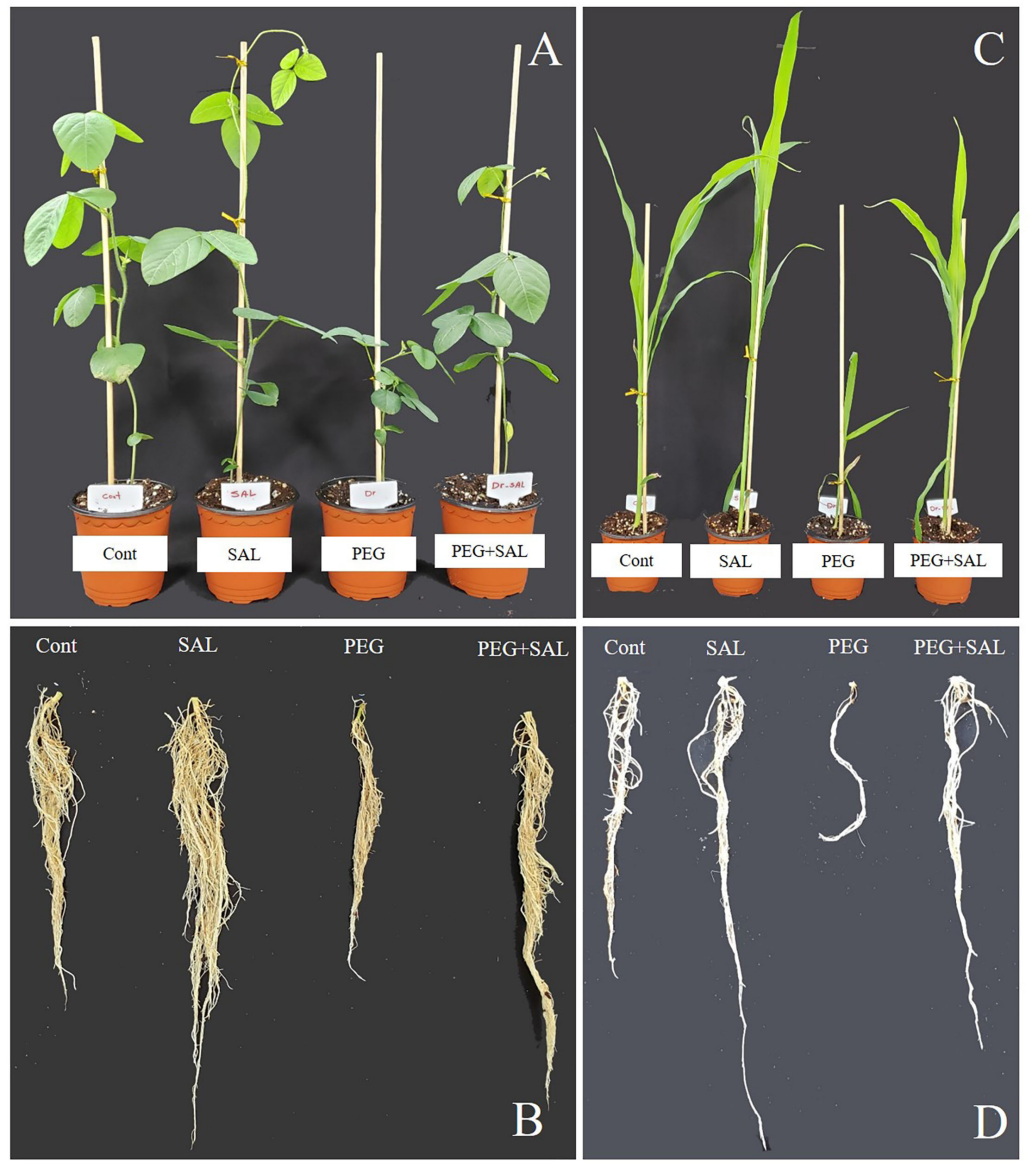
Impact of salvianolic acid on soybean and maize plant growth and root under normal and stress conditions 8 days post-treatment **(A–D)**. Treatments: Cont (control), SAL (0.1 μM salvianolic acid), SAL (1 μM salvianolic acid), PEG (25% polyethylene glycol), SAL (0.1 μM salvianolic acid) + PEG (25% polyethylene glycol), and SAL (1 μM salvianolic acid) + PEG (25% polyethylene glycol). Values show the means ± SE (*n* = 6) and significant differences at *p* < 0.05 (Tukey test).

**Figure 2:**
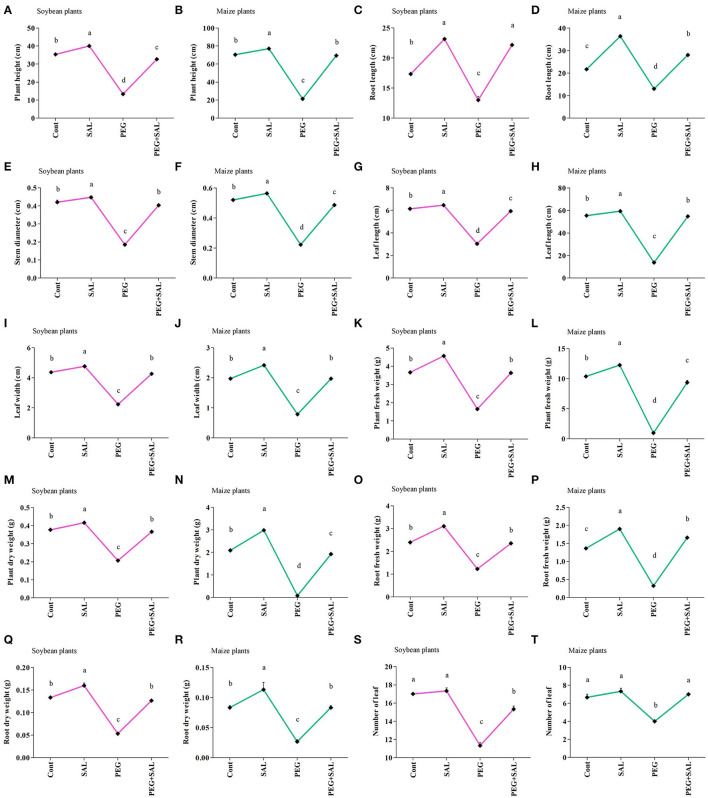
Impact of salvianolic acid on soybean and maize plant growth parameters under normal and stress conditions 8 days post-treatment **(A–T)**. Treatments: Cont (control), SAL (0.1 μM salvianolic acid), SAL (1 μM salvianolic acid), PEG (25% polyethylene glycol), SAL (0.1 μM salvianolic acid) + PEG (25% polyethylene glycol), and SAL (1 μM salvianolic acid) + PEG (25% polyethylene glycol). Values show the mean ± SE (*n* = 6) and significant differences at *p* < 0.05 (Tukey's test). Bars with different lowercase letters are significantly different from each other.

The impact of the salvianolic acid (SAL) on the growth of soybean and maize seedlings under osmotic stress conditions and without stress was investigated in pot trials. This detrimental abiotic stress factor negatively affected growth attributes (Figures 1A–D, 2A–T) in unstressed and untreated soybean and maize plants. Conversely, these growth attributes were promoted in SAL-treated plants under stress. For instance, soybean plant height and root length were promoted by 59.18 and 41.33% in the osmotic stress treatments, respectively. Similarly, in SAL-treated soybean plants, plant fresh weight and root fresh weight were elevated by 54.54 and 47.65% in the osmotic stress treatment, respectively, when compared to the control plants (Figures 2K,O).

#### Photosynthetic Pigments

**Figure 3:**
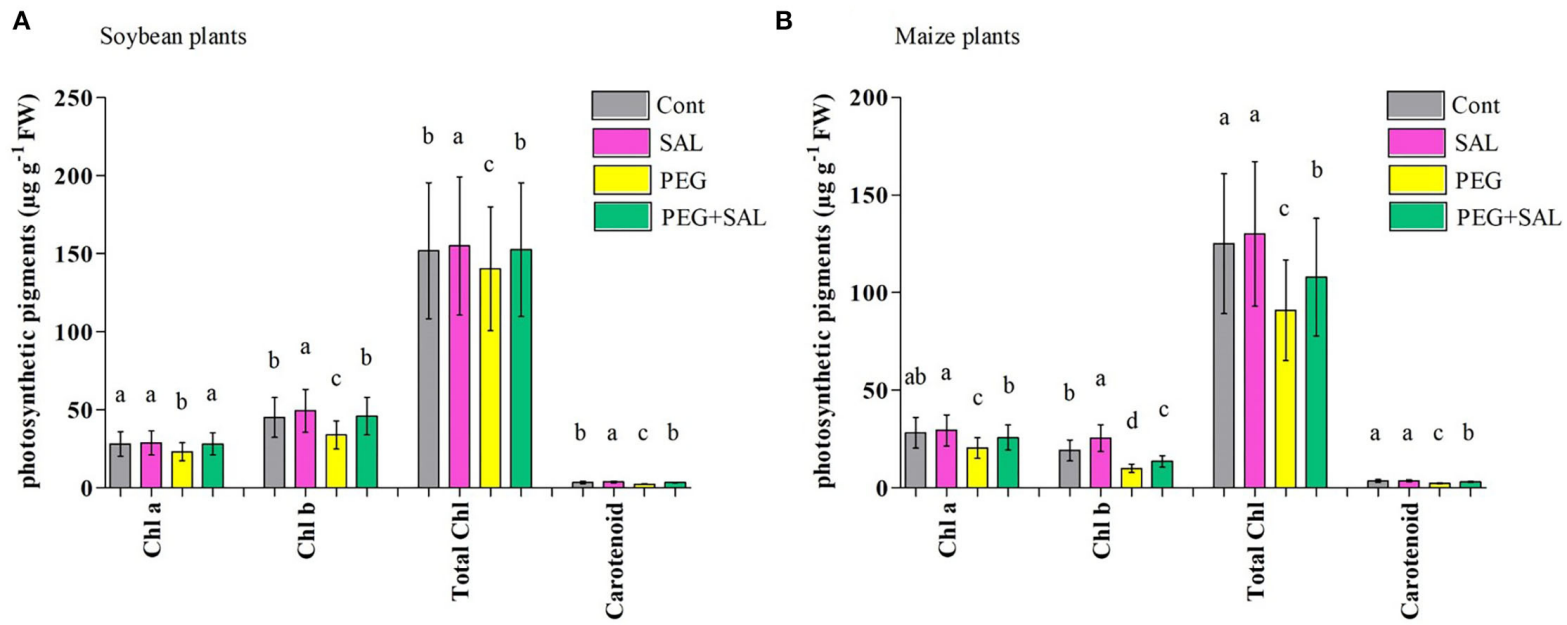
Effect of salvianolic acid application on soybean and maize plant photosynthetic parameters “chlorophyll a, Chla; chlorophyll b, Chlb; total chlorophyll, total Chl; and carotenoid contents” **(A,B)**. Treatments: Cont (control), SAL (0.1 μM salvianolic acid), SAL (1 μM salvianolic acid), PEG (25% polyethylene glycol), SAL (0.1 μM salvianolic acid) + PEG (25% polyethylene glycol), and SAL (1 μM salvianolic acid) + PEG (25% polyethylene glycol). Values show the mean ± SE (*n* = 6) and significant differences at *p* < 0.05 (Tukey's test). Bars with different lowercase letters are significantly different from each other.

Our results depicted that Chla, Chlb, and carotenoid contents were significantly higher in the SAL-treated stressed plants. Similarly, increased total chlorophyll content (TCC) was observed in SAL-treated stressed plants (Figures 3A,B). A decline in TCC was perceived in osmotic-stressed soybean and maize plants (7.56 and 27.28%, correspondingly). On the other hand, SAL application contributed to an 8.02% (soybean) and 15.75% (maize) promotion in TCC under osmotic stress conditions when compared to the stressed control plants, and the difference was significant (*p* < 0.05; Figures 3A,B).

#### Amino Acid Accumulation

**Figure 4:**
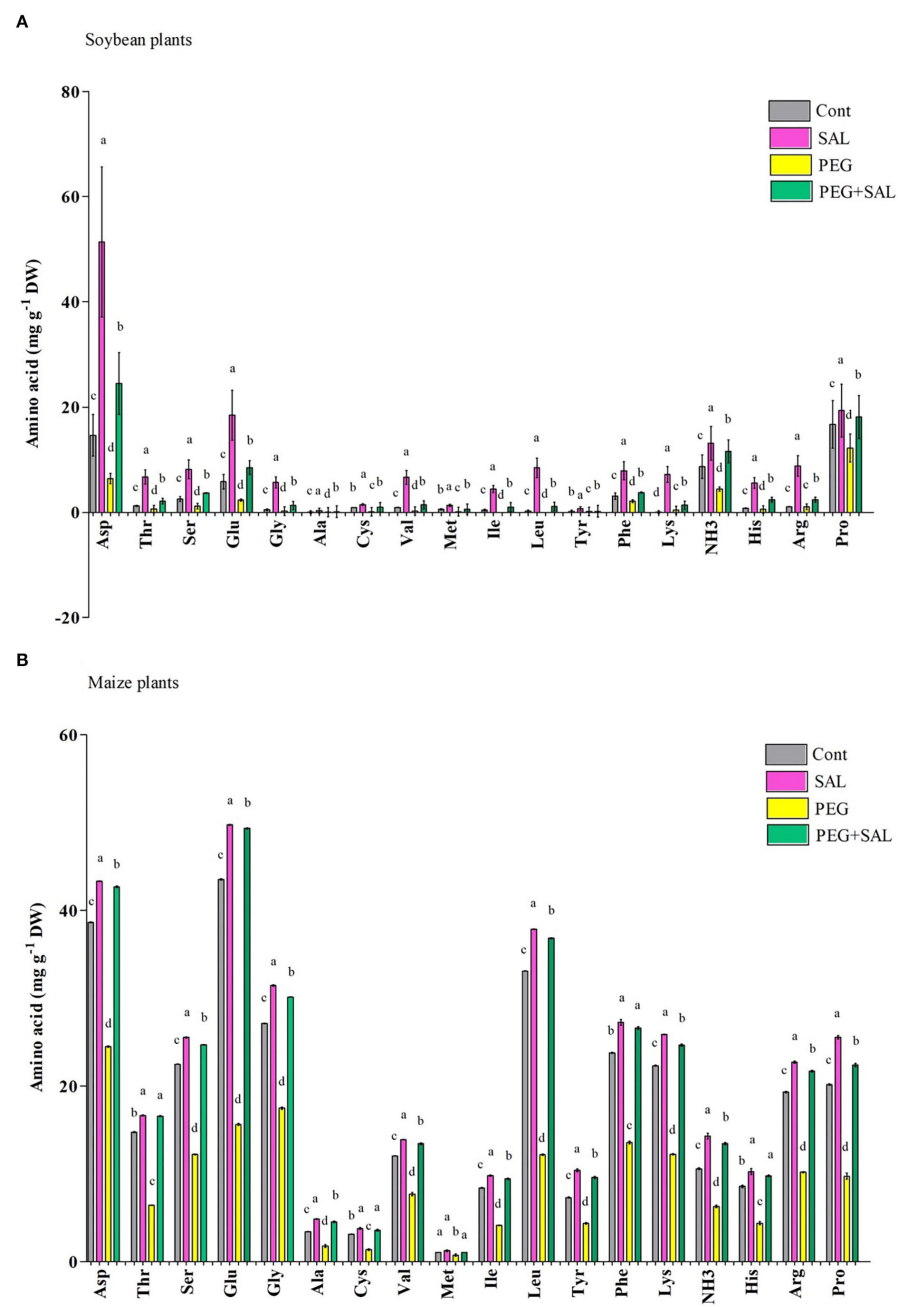
Effect of salvianolic acid application on amino acid content in leaves of soybean and maize grown under normal and stress conditions 8 days post-treatment **(A,B)**. Treatments: Cont (control), SAL (0.1 μM salvianolic acid), SAL (1 μM salvianolic acid), PEG (25% polyethylene glycol), SAL (0.1 μM salvianolic acid) + PEG (25% polyethylene glycol), and SAL (1 μM salvianolic acid) + PEG (25% polyethylene glycol). Values show the mean ± SE (*n* = 6) and significant differences at *p* < 0.05 (Tukey's test). Bars with different lowercase letters are significantly different from each other.

Eighteen amino acids were identified in soybean and maize seedlings exposed to various treatments (Figures 4A,B). Osmotic stress caused a noticeable decrease in amino acid content in maize and soybean seedlings over 8 days. Proline content decreased by 26.91% (soybean) and 51.74% (maize) in osmotic-stressed plants, correspondingly. In contrast, plants treated with SAL had increased proline content (soybean, 32.66%; maize, 56.49%). Moreover, asparagine was the highest in osmotic-stressed plants 8 days after the application of SAL on the impaired plants, while alanine and tyrosine were the lowest in control and stressed soybean seedlings (Figure 4A).

#### Salvianolic Acid Regulates H_2_O_2_, MDA, and Fatty Acid Content

**Figure 5:**
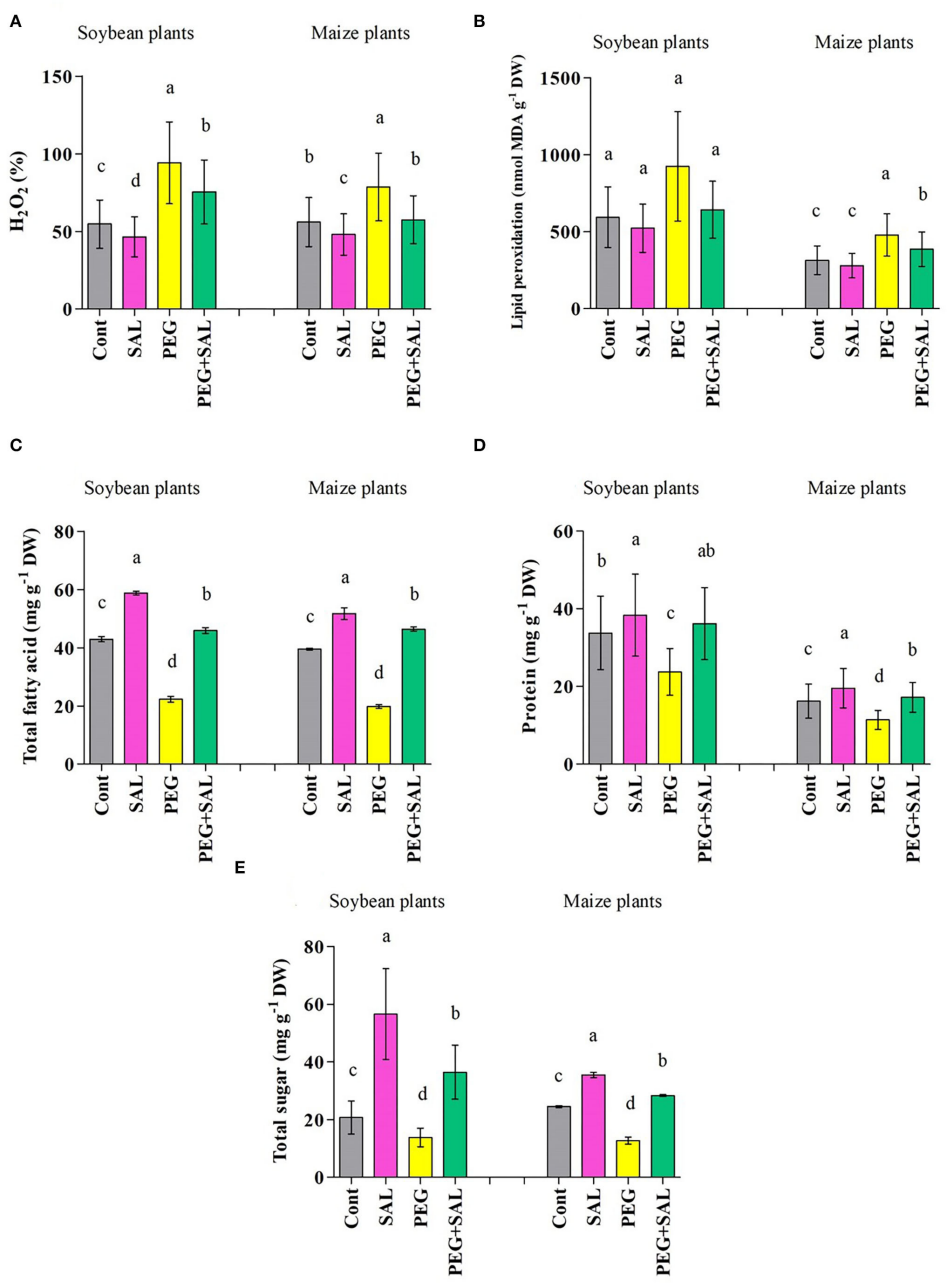
**(A)** H_2_O_2_, **(B)** MDA, **(C)** total fatty acid, **(D)** protein, and **(E)** sugar content in leaves of soybean and maize grown under normal and stress conditions and treated with salvianolic acid for 8 days (8DAT). Treatments: Cont (control), SAL (0.1 μM salvianolic acid), SAL (1 μM salvianolic acid), PEG (25% polyethylene glycol), SAL (0.1 μM salvianolic acid) + PEG (25% polyethylene glycol), and SAL (1 μM salvianolic acid) + PEG (25% polyethylene glycol). Values show the mean ± SE (*n* = 6) and significant differences at *p* < 0.05 (Tukey's test). Bars with different lowercase letters are significantly different from each other.

The H_2_O_2_ content was elevated by 41.83 and 28.76% in soybean and maize plants under osmotic stress, correspondingly (Figure 5A). Utmost mitigations of 19.89 and 26.77% in H_2_O_2_ content were recorded in SAL-treated soybean and maize plants under osmotic stress, correspondingly (*p* < 0.05).

As exhibited in Figure 5B, stress conditions elevated the generation of malondialdehyde (MDA) in the untreated soybean (35.81%) and maize (34.45%) plants. SAL treatment ameliorated MDA formation in osmotic-stressed plants by 30.46% (soybean) and 19.30% (maize) (*p* < 0.05).

The total fatty acid content in soybean and maize seedlings diminished in response to osmotic stress (soybean, 48.01% and maize, 49.87%) (Figure 5C). However, SAL-treated plants exhibited higher fatty acid content under stress and no stress conditions (soybean, 51.41% and maize, 57.32%).

#### Protein and Sugar Synthesis

We observed an increase in protein values in soybean (11.85%) and maize (17%) upon SAL application when compared to the untreated plants. However, the values were found to be diminished by 29.74% (soybean) and 29.82% (maize) under osmotic stress (Figure 5D). Under stress conditions, SAL treatment resulted in higher protein content (soybean, 34.35% and maize, 33.82%).

Stress resulted in a marked decline in the sugar content in soybean (33.43%) and maize (47.97%) plants (Figure 5E). SAL application increased the sugar content by 62.07% (soybean) and 55.05% (maize) under osmotic stress conditions (Figure 5E).

#### Antioxidant Assay

**Figure 6:**
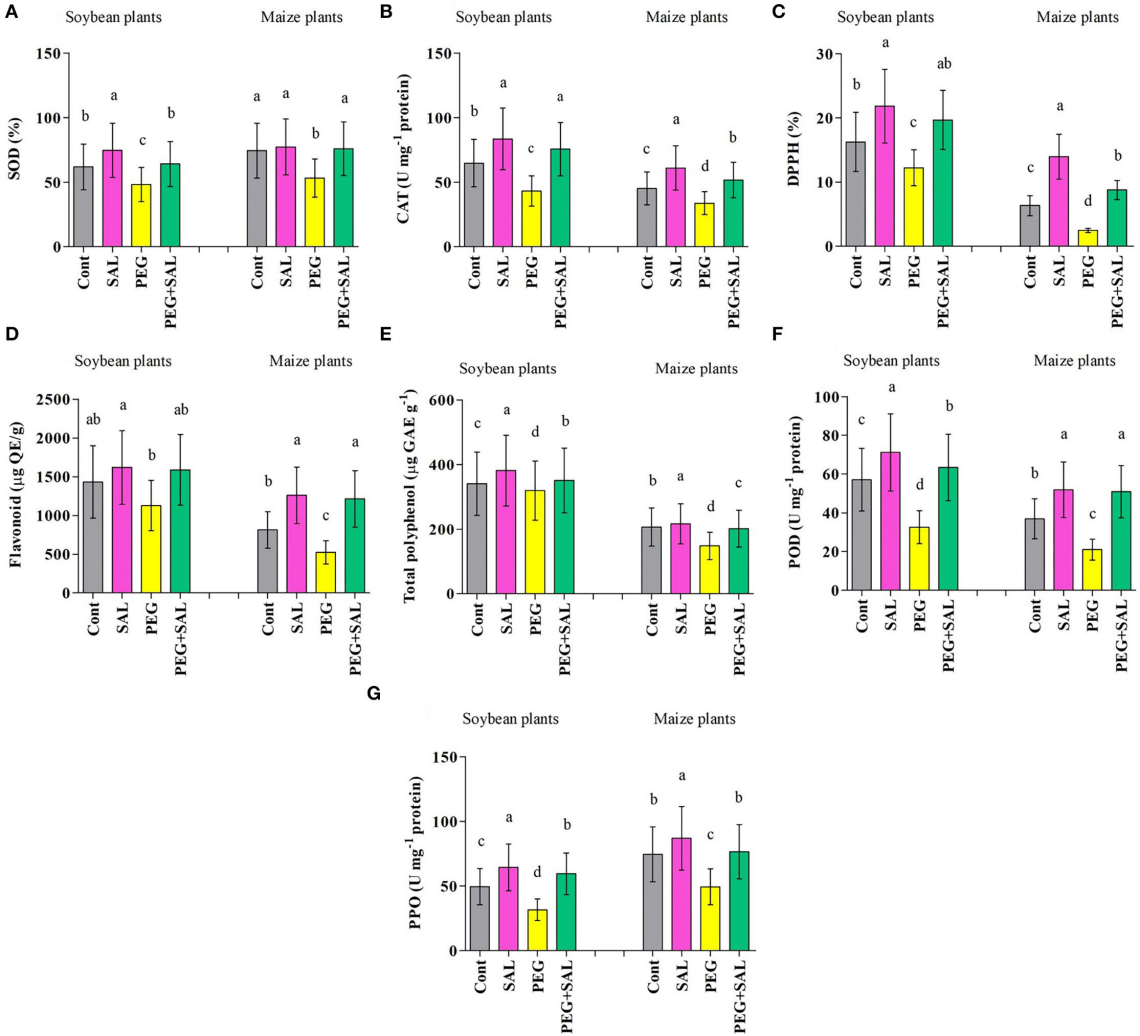
Effect of salvianolic acid application on antioxidant content “SOD **(A)**; CAT **(B)**; DPPH **(C)**; Flavonoids **(D)**; Total polyphenol **(E)**; POD **(F)**; PPO **(G)**” of soybean and maize leaves grown under normal and stress conditions 8 days post-treatment. Treatments: Cont (control), SAL (0.1 μM salvianolic acid), SAL (1 μM salvianolic acid), PEG (25% polyethylene glycol), SAL (0.1 μM salvianolic acid) + PEG (25% polyethylene glycol), and SAL (1 μM salvianolic acid) + PEG (25% polyethylene glycol). Values show the mean ± SE (*n* = 6) and significant differences at *p* < 0.05 (Tukey's test). Bars with different lowercase letters are significantly different from each other.

Stress resulted in a decrease in enzymatic and non-enzymatic antioxidant functions (SOD, CAT, DPPH, flavonoid, total polyphenol, POD, and PPO) in soybean and maize plants. SAL application distinctly upraised antioxidant activities under stress conditions (Figures 6A–G). For instance, DPPH and total polyphenol activity were higher in SAL-treated soybean (DPPH, 37.86% and total polyphenol, 9%) and maize (DPPH, 71.88% and total polyphenol, 26.46%) plants affected by osmotic stress in comparison to untreated stressed plants (Figures 6A,B).

#### Characterization of Nutrient Content in Plants

The nutrients like Ca, K, and P were inspected in soybean and maize plants to investigate the impact of the salvianolic acid on the nutrient value of soybean and maize plants and its recovery function (Table 2). In unstressed plants, a rise was observed in Ca, K, and P in plants treated with salvianolic acid compared to the control plants. Plant nutrients were modulated in SAL-treated stressed plants, which indicated enhancement in *K*- and *P*-values and a reduction in the Ca value under unfavorable conditions.

**Table 2:** Macronutrient accumulation in soybean and maize plants grown under stress and control conditions with or without salvianolic acid.

**Sample name**	**Ca (μg/kg)**	**K (μg/kg)**	**P (μg/kg)**
**8DAT**			
**Soybean**			
Cont	6.55 ± 0.2bc	41.18 ± 0.59c	5.26 ± 0.10c
SAL	7.27 ± 0.15a	49.94 ± 0.97b	6.79 ± 0.04a
PEG	6.77 ± 0.11ab	50.18 ± 0.09b	5.29 ± 0.09c
PEG+SAL	6.14 ± 0.03c	55.84 ± 0.92a	6.30 ± 0.20b
**Maize**			
Cont	8.31 ± 0.06bc	61.18 ± 0.59b	7.37 ± 0.16b
SAL	9.28 ± 0.14a	64.93 ± 4.02ab	8.40 ± 0.06a
PEG	8.61 ± 0.06b	65.18 ± 0.09ab	7.40 ± 0.04b
PEG+SAL	8.18 ± 0.01c	69.34 ± 0.58a	8.21 ± 0.11a

*Values show the mean ± SE (n = 6). Data within the same column and different lowercase letters are significantly different at p < 0.05 (Tukey's test)*.

#### Salvianolic Acid Altered the Expression Pattern of Osmotic Stress-Responsive Genes in Soybean and Maize

Twelve genes were examined for their transcript patterns in soybean and maize plants under SAL application and abiotic stress conditions.

#### Association Analysis of Stress-Responsive Genes in Soybean

**Figure 7:**
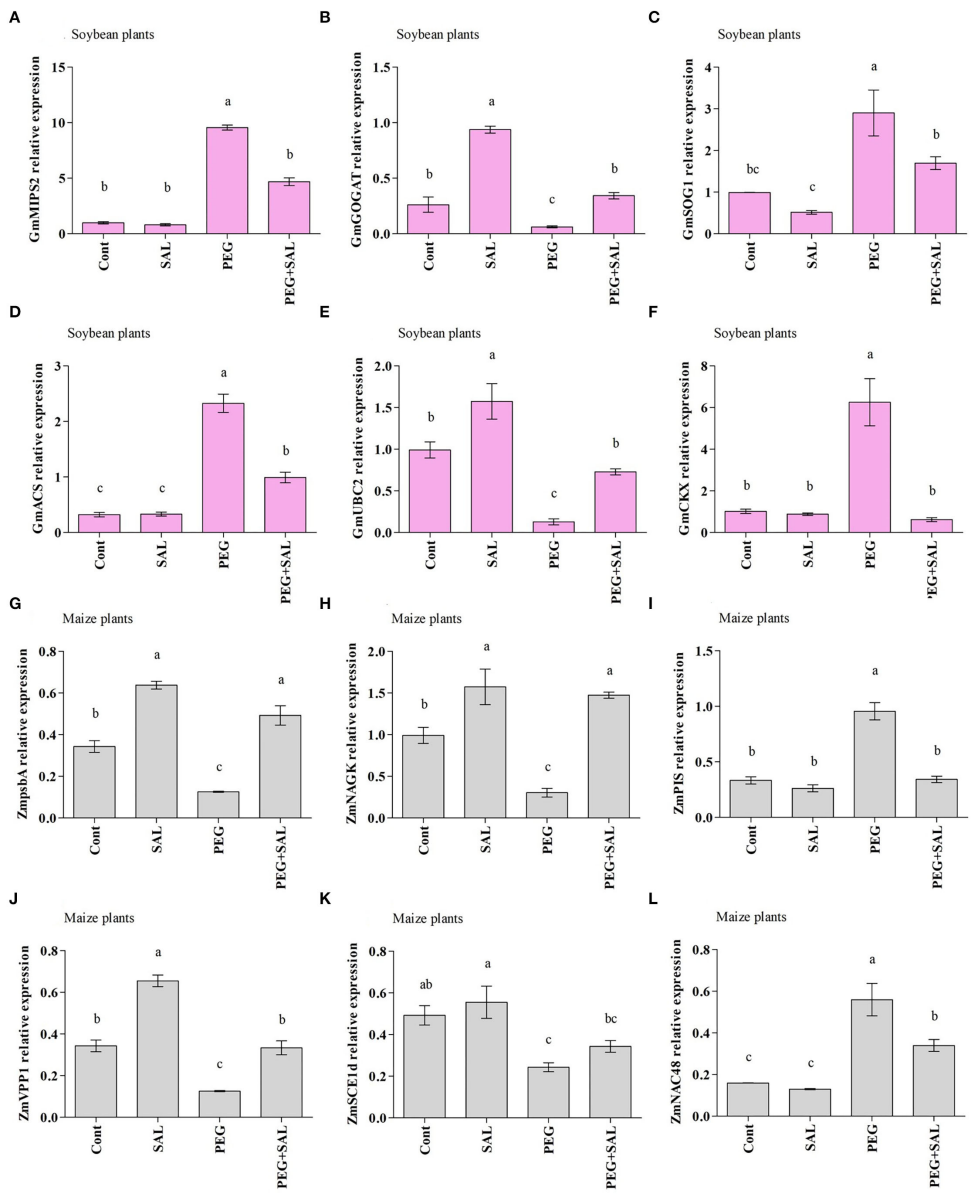
Real-time expression analysis of *GmMIPS2* **(A)**, *GmGOGAT* **(B)**, *GmSOG1* **(C)**, *GmACS* **(D)**, *GmUBC2* **(E)**, *GmCKX* **(F)**, *ZmpsbA* **(G)**, *ZmNAGK* **(H)**, *ZmPIS* **(I)**, *ZmVPP1* **(J)**, *ZmSCE1d* **(K)**, and *ZmNAC48* **(L)** in leaves of soybean and maize plants grown under normal and stress conditions and treated with salvianolic acid for 8 days (8DAT). Treatments: Cont (control), SAL (0.1 μM salvianolic acid), SAL (1 μM salvianolic acid), PEG (25% polyethylene glycol), SAL (0.1 μM salvianolic acid) + PEG (25% polyethylene glycol), and SAL (1 μM salvianolic acid) + PEG (25% polyethylene glycol). Values show the mean ± SE (*n* = 6) and significant differences at *p* < 0.05 (Tukey's test). Bars with different lowercase letters are significantly different from each other.

This study characterized the gene *GmMIPS2* in soybean, which showed contrasting responses under SAL application and abiotic stress conditions. An increase in *GmMIPS2* expression levels was found in osmotic-stressed plants. However, SAL treatment of osmotic-stressed plants decreased the expression by 50.94%, in contrast to that observed in the untreated plants (Figure 7A).

The stress diminished *GOGAT* gene expression in soybean plants, while SAL treatment elevated the expression of this gene (Figure 7B). For instance, the SAL application enhanced *GmGOGAT* gene expression by 81.90%, under osmotic stress.

The effect of osmotic stress and SAL application on *SOG1* was examined through the modification of the *SOG1* gene expression (*GmSOG1*) (Figure 7C). Differences were observed in the expression of the *GmSOG1* gene in control and SAL-treated plants under stress conditions. Furthermore, improved *GmSOG1* expression was noticed in the stressed plants. SAL-treated plants revealed a decline in *GmSOG1* expression in contrast to untreated stressed plants (41.59% under stress conditions).

An increase in *GmACS* expression was discerned in osmotic-stressed plants (86.13%) in comparison to a decrease in SAL-treated stressed plants, which decreased by 57.37% (Figure 7D).

As illustrated in Figure 7E, osmotic-stressed plants exhibited an 87.04% reduction in *GmUBC2* expression when compared to the control plants. On the other hand, improved expression of *GmUBC2* was perceived in SAL-treated plants exposed to abiotic stress. SAL treatment increased *GmSAP16* expression by 82.34% under osmotic stress (Figure 7E).

Abiotic stress increased *GmCKX* expression by 83.74% (Figure 7F), but SAL treatment decreased *GmCKX* expression by 90.07%.

#### Association Analysis of Stress-Responsive Genes in Maize

The *ZmpsbA* transcript pattern in maize seedlings under abiotic stress and SAL treatment is exhibited in Figure 7G. A decline in *ZmpsbA* expression was discerned in osmotic-stressed plants by 63.42%. On the other hand, SAL treatment upraised the *ZmpsbA* expression in osmotic-stressed soybean plants by 74.52%.

As shown in Figure 7H, the abiotic stress reduced *ZmNAGK* expression in untreated stressed plants, but the expression increased by 79.36% in SAL-treated stressed plants (Figure 7H).

In the current experiment, we assessed the *ZmPIS* transcription pattern in maize plants. An increase in *ZmPIS* expression level was recorded in osmotic-stressed plants when compared to the expression in the unstressed plants. Although *ZmPIS* expression level increased in osmotic-stressed plants, the SAL implementation declined the expression by 64.06% (Figure 7I).

The effects of abiotic stress and SAL application on the expression of *ZmVPP1* were evaluated in maize seedlings through modification in the *VPP1* gene expression (*ZmVPP1*) (Figure 7J). During unstressed conditions, variations were shown in the *ZmVPP1* gene expression in control and SAL-treated plants; conversely, declined *ZmVPP1* expression was perceived in stressed plants. SAL-treated plants showed a rise (62.41%) in *ZmVPP1*expression.

The findings of the current study demonstrated a reduced *ZmSCE1d* expression in maize plants under osmotic stress conditions in contrast to the control plants. The association of maize plants with SAL noticeably enhanced the upregulation of *SCE1d*. A higher *ZmSCE1d* expression level (29.12%) was exhibited in SAL-treated stressed plants (Figure 7K).

We examined the expression pattern of *ZmNAC48* under normal and stress conditions. Enhanced *ZmNAC48* expression was discerned in maize seedlings affected by abiotic stress. The *ZmNAC48* expression level increased by 71.42% under osmotic stress (Figure 7L). In addition, SAL-treated maize plants depicted reduced *ZmNAC48* expression under stress conditions. Under SAL application, osmotic-stressed plants exhibited 39.28% lower *ZmNAC48* expression, compared to the untreated stressed plants.

## Discussion

To ascertain the impacts of exogenously applied salvianolic acid (SAL) on osmotic stress, we examined whether the irrigation of soybean and maize seedlings with SAL would mitigate the symptoms of osmotic stress. SAL-treated seedlings (0.1 and 1 μM) seemed to be healthier, and we perceived that SAL, when applied to the soil, enhances plant growth and development. Additionally, it enhances osmotic stress tolerance and delays foliar wilting, which is evident from the improved growth attributes. Our data showed that treatment of unstressed and stressed plants with SAL results in increased height, root length, leaf area, chlorophyll and carotenoid values, K and P contents, etc. Additionally, the K and P content of osmotic-stressed plants treated with SAL was higher than that of untreated stressed plants. These functions are presumably achieved through amelioration processes associated with photosynthesis and other metabolisms.

Photosystem II consists of a multi-protein complex (D1 and D2 proteins) and performs a function in the oxygen-evolving photosynthetic organisms. The D1 protein is an essential constituent of oxygenic photosynthesis in plants. The *psbA* (encoding D1 protein) has a vital role in protecting photosystem II (PSII) from oxidative damage in plants (Nelson and Yocum, 2006; Mulo et al., 2012). Here, we found that SAL application improved *ZmpsbA* expression, which leads to enhanced D1 protein and confers osmotic stress endurance in maize plants. A study conducted by Huo et al. (2016) demonstrated that enhanced *ZmpsbA* expression, along with higher antioxidant enzyme activities, reduced hydrogen peroxide, malondialdehyde, and ion leakage during osmotic stress, suggesting the role of overexpressed D1 in removing immoderate ROS and boosting antioxidant capability.

Vacuolar H^+^-pyrophosphatase has an important function in plant response to osmotic stress (Kriegel et al., 2015). The expression of its encoding gene (*VP1*) is prevalent in varied tissues (Gamboa et al., 2013; Yang et al., 2015), and its overexpression can improve crop tolerance to drought and salinity (Anjaneyulu et al., 2014; Schilling et al., 2014; Lv et al., 2015; Wang et al., 2016). In the present study, the expression level of *ZmVPP1* is rapidly upregulated in stressed maize plants upon SAL application, which proves the stress alleviation effect of SAL. Wang et al. (2016) reported that maize with increased *ZmVPP1* expression shows enhanced drought endurance. They believed that it is most probably due to improved photosynthetic efficiency and root development.

The plant-specific NAC gene family (NAC, ATAF, and CUC) encodes one of the biggest family of transcription factors and are extensively distributed in a wide range of plants (Olsen et al., 2005). Several NAC members have been practically distinguished in the developmental programs (Souer et al., 1996; Aida et al., 1997; Takada et al., 2001; Weir et al., 2004), growth hormone signaling (Xie et al., 2000; Fujita et al., 2004), defense (Collinge and Boller, 2001; Hegedus et al., 2003), and leaf senescence (Fraga et al., 2021). Various NAC genes participate in reactions to abiotic stresses, which include flood, water deficit, salinity, and cold (Fujita et al., 2004; Olsen et al., 2005; Hu et al., 2006; He et al., 2017; Gao et al., 2020, 2021; Mao et al., 2021). Enhanced expression of *ZmNAC48* was detected in maize plants subjected to osmotic stress. Mao et al. (2021) demonstrated that overexpressing *ZmNAC48* enhanced drought endurance, regulated ABA biosynthesis, reduced water loss, and improved stomatal closure. Therefore, we speculate that *ZmNAC48* expression is reduced in SAL-treated stressed plants due to the stress-relieving effect of SAL.

Phospholipids are one of the major structural components of membranes. They also function as signaling precursors or second messengers to modulate plant growth, development, and adaptation to environmental change (Xue et al., 2009). Phosphatidylinositol is a precursor of inositol-containing phospholipids in plant cells and phosphatidylinositol synthase (*ZmPIS*) is a main enzyme in the phospholipid pathway and accelerates the development of phosphatidylinositol (Liu et al., 2013). Here, we noticed overexpression of the *ZmPIS* in maize plants subjected to osmotic stress conditions. On the other hand, the transcript level of *ZmPIS* gene was altered in stressed maize plants under SAL treatment. Liu et al. (2013) reported that *ZmPIS* modulates the plant response to drought stress via modifying membrane lipid composition and enhancing ABA synthesis in maize. Thus, it could be presumed that *ZmPIS* is engaged in plant responses to osmotic stress in maize.

Phytohormones play essential roles in a broad range of physiological processes (Gerashchenkov and Rozhnova, 2013). Ethylene is a multifunctional plant hormone with varied functions, including germination, growth, cell elongation, fruit ripening, and senescence (Iqbal et al., 2017). Previous reports have confirmed the effect of abiotic stress on phytohormone performance, including ethylene (Habben et al., 2014; Riyazuddin et al., 2020). The findings of the current survey imply that osmotic stress enhances the expression level of ethylene-related gene (ACS). It has been proved that under water deficit conditions, ethylene caused leaf abscission and subsequently minimized water loss (Arraes et al., 2015). Our study shows that the application of SAL boosts soybean seedlings to confront stressful environments by diminishing the ethylene content.

Plants generally trigger a vast variety of defense mechanisms to improve endurance to water deficit conditions. Cytokinins (CKs) help to modulate plant development and conciliate plant endurance to water deficit stress. CK oxidases/dehydrogenases (CKXs) help to control CK metabolism. Growing evidence indicated that CKXs have an important role in diverse plant physiological and developmental alterations under stress (Pospíšilov et al., 2016; Hai et al., 2020). Our findings demonstrated that the expression of *CKX* was highly responsive to osmotic stress and was upregulated by dehydration. This was consistent with previous findings (Le et al., 2012). Moreover, we observed a reduction in *CKX* expression level in SAL-treated stressed plants. An enhancement in CK content was also demonstrated to promote leaf longevity and photosynthesis under drought stress, consequently improving water deficit tolerance without yield penalties (Rivero et al., 2007; Peleg et al., 2011). Our analysis of *CKX* has provided insights into CK metabolism in soybean under SAL application and osmotic stress conditions.

Ubiquitination is an essential kind of post-translational alteration of proteins observed in all eukaryotes. Ubiquitination modulates vital biological processes, including plant growth processes, photomorphogenesis, vascular differentiation, flower development, DNA repair, and biotic and abiotic stress factors (Dreher and Callis, 2007). The ubiquitin-conjugating enzyme, *UBC*, is a key enzyme that is involved in ubiquitination. A previous study demonstrated that *GmUBC2* is involved in the leaf development, oxidative stress responses, ion homeostasis modulation, osmolyte synthesis, and stress tolerance responses (Zhou et al., 2010; Zhiguo et al., 2015). Our findings have shown that *GmUBC2* is overexpressed in SAL-treated plants under stress conditions, which provides endurance to osmotic stress.

Reactive oxygen species (ROS) have a vital role in the adaptation mechanism of plants to unfavorable conditions (Huang et al., 2019). ROS at higher concentrations can cause oxidative damage, deteriorate membranes and proteins, disrupt metabolic activities, initiate programmed cell death, and debilitate enzymes (Choudhury et al., 2017). We detected higher H_2_O_2_ and MDA contents in stressed plants, which could be due to an imbalance in the rate of ROS production and removal (Huang et al., 2019). In contrast, SAL application evidently diminished the augmented H_2_O_2_ and MDA concentrations in osmotic-stressed plants toward the end of the inspection. Therefore, SAL might suppress the formation of ROS and thus inhibit oxidative-induced plasma membrane deterioration under abiotic stress (Sewelam et al., 2016; Castro et al., 2021).

Myo-inositol phosphate synthase (MIPS) is a central molecule needed for many processes, including cell metabolism, plant growth and development, and cell wall biogenesis (Loewus and Murthy, 2000; Meng et al., 2009). It has been demonstrated that myo-inositol is a key factor that ascertains whether oxidative stress activates or prevents defense responses during cell death provoked by hydrogen peroxide (Chaouch and Noctor, 2010). We found that the transcript levels of *GmMIPS2* increased in soybeans cultivated under osmotic stress conditions. A study conducted by Ishibashi et al. (2011) showed that *GmMIPS2* is involved in drought stress signaling via ROS formation caused by drought stress.

As a crucial controlling process of post-translational alterations, Sumoylation have an important role in plants in developmental, hormonal, and environmental stress responses (Park and Yun, 2013; Wang et al., 2020). *ZmSCE1d*, a maize class-I SUMO conjugating enzyme, has been stated to take part in salt and drought tolerance activities (Wang et al., 2019). A previous study by Wang et al. (2020) reported that overexpression of *ZmSCE1d* enhanced SUMO conjugates and promoted drought endurance in plants. Taken together, our data showed that *ZmSCE1d* overexpression enhanced osmotic stress tolerance and antioxidant capability in maize plants under SAL treatment.

Land plants are exposed to hostile environments that suppress their growth and productivity. Therefore, they have evolved mechanisms to evade or endure adverse environmental conditions (He and Ding, 2020). It has been shown that environmental factors, including drought, salinity, and cold, cause alterations in the fatty acid content (Sui et al., 2018). Fatty acids control the concentration of ROS by specifically influencing the ROS-generating enzymes (Lim et al., 2017). The results displayed that osmotic stress affects fatty acid levels. The reduced fatty acid level was observed in soybean and maize seedlings under osmotic stress. Conversely, SAL-treated seedlings were less affected by this stress and exhibited a remarkably higher level of fatty acids. Singh et al. (2020) indicated that fatty acids boosted drought and salinity stress tolerance in soybean plants. Therefore, these data suggest that SAL utilization may induce the activation of phospholipases and phospholipid-derived molecules, which are engaged in plant protection mechanisms (Hou et al., 2016).

Plants have evolved intricate strategies to respond to stress via modifications at physiological and molecular levels (Qi et al., 2018). Accumulation of ROS in plants leads to DNA damage. One mechanism to combat oxidative damage and minimize immoderate ROS aggregation is via inner defensive mechanisms that entail antioxidant functions (Agarwal and Pandey, 2004; Gill and Tuteja, 2010). Besides the antioxidation pathway, plants have developed an effective system recognized as the DNA damage response (DDR) pathway (Baxter et al., 2013; Poku et al., 2021). A plant-specific transcription factor, the suppressor of gamma response 1 (*SOG1*) gene, has been identified as a major gene in plant response to DNA damage (Yoshiyama et al., 2009). The results of the present study revealed that the antioxidant function and *SOG1* expression level diminished in osmotic-stressed plants, while this function and expression pattern was enhanced upon SAL application in the stressed plants. A previous study implied that the expression of antioxidant enzymes could be triggered or hindered under abiotic stresses (Zhu et al., 2004). Moreover, it has been confirmed that SOG1 overexpression led to a higher survival rate and antioxidant accumulation in osmotic-stressed plants (Poku et al., 2021). This increase in antioxidant activity and SOG1 expression level suggests that SAL upraises the capability to scavenge excessive ROS, diminishes oxidative damage, and promotes osmotic stress endurance.

Deleterious environmental situations can severely alter sugar concentration in leaves. Sugar modulates various physiological functions, such as photosynthesis, osmotic homeostasis, and protein and lipid metabolism (Martínez-Noël and Tognetti, 2018). It has been confirmed that sugars may act as osmotically active molecules or protective agents upon membranes and enhance plant endurance under deleterious conditions (Sánchez et al., 1998; Sami et al., 2016). Our findings depicted an obvious aggregation of sugars in soybean and maize plants irrigated with SAL under normal and osmotic stress conditions. Saddhe et al. (2021) attested that sugar elevation also improves proline concentration under stress conditions. Considering the present study, the SAL treatment led to further sugar aggregation, which served as an osmoprotectant to modulate osmotic alterations, maintain membrane functions, and improve recovery from osmotic stress.

Previous studies have demonstrated that modulation of nitrogen metabolism is strictly linked to drought stress responses in plants (Zhong et al., 2018, 2019). Nitrogen is a fundamental element for crop growth, development, and yield and is involved in many physiological processes. Nitrogen in the form of ${\text{NH}}_{4}^{+}$ is changed to glutamate and glutamine via the glutamine synthetase, glutamate synthetase (GOGAT), and glutamate dehydrogenase pathways (Xu and Zhou, 2006). Considering that the augmentation of ${\text{NH}}_{4}^{+}$ in the process of nitrogen metabolism is harmful to plant cells (Nguyen et al., 2005), sustaining the activities of enzymes, such as GOGAT, in the nitrogen metabolism process is vital for plant growth (Du et al., 2020a). GOGAT activity is linked to drought stress response, and it is usually regarded as a metabolic indicator of drought tolerance (Nagy et al., 2013; Singh and Ghosh, 2013). In this experiment, osmotic stress conditions reduced the activity of GOGAT, which was in agreement with a previous study (Nagy et al., 2013). This reduced enzyme activity directly affects the efficiency of N uptake and utilization. On the other hand, SAL application enhanced GOGAT activity under osmotic stress conditions. This finding suggests that SAL assists in maintaining N metabolism and enhancing adaptation to osmotic stress.

Amino acids participate in the synthesis of numerous plant products that confer plant responses to adverse conditions (Batista-Silva et al., 2019). In this study, the amino acid content was elevated in soybean and maize plants subjected to osmotic stress conditions, which was in harmony with previous studies (Batista-Silva et al., 2019; Li et al., 2019; Trovato et al., 2021). This amino acid aggregation may be engaged in osmotic acclimatization, ROS scavenging, and protein maintenance (Wu et al., 2014). The SAL treatment recovered amino acid value in the stressed plants throughout the restoration time. A rise in proline concentration was discerned in stressed soybean and maize plants, which was consistent with the studies on varied plants (Khattab, 2007; Bassuony et al., 2008). Proline serves as a reactive oxygen species scavenger, stabilizes membrane and protein structure, minimizes cell damage, and enhances the tolerance of plants toward environmental changes (Teixeira et al., 2020). In addition, proline also functions as a nutritional repository that can be consumed throughout the revival stage to help plants withstand environmental crises (Heinemann and Hildebrandt, 2021). In this conducted research, SAL treatment caused augmentation of amino acids, distinctly proline content, in stressed plants. This SAL-induced proline accumulation might be relevant to adaptive tactics that ameliorate osmotic acclimatization during osmotic stress.

Nitric oxide participates in numerous physiological processes in plants, such as growth, hormone responses, antioxidant activities, defense reactions, and abiotic stress responses (Peng et al., 2016; Hasanuzzaman et al., 2018; Su et al., 2018). It has been demonstrated that arginine (Arg) is a crucial precursor of NO (Winter et al., 2015). Since *N*-acetylglutamate kinase (NAGK) takes part in arginine biosynthesis, the increased osmotic stress endurance in the plants expressing *ZmNAGK* could be due to the NO augmentation through the arginine metabolism pathway (Huang et al., 2017). In this study, we observed enhanced expression of *ZmNAGK* in SAL-treated stressed plants. Furthermore, maize plants overexpressing *ZmNAGK* aggregated more arginine in response to SAL treatment under osmotic stress conditions. Liu et al. (2019) reported that enhanced expression of the *ZmNAGK* gene promoted drought endurance via greater water preservation, antioxidant defense capability, less oxidative damage, and aggregation of more arginine.

## Conclusion

In summary, through this study, we have exhibited that salvianolic acid (SAL) can remarkably improve soybean and maize vitality and tolerance under osmotic stress conditions. The potential of SAL under abiotic caused stress modulated host growth by mitigating osmotic stress in maize and soybean plant. Furthermore, SAL application amended host biochemistry to lessen the drastic effects of osmotic stress. In this survey, osmotic stress restrained several genes, while SAL was able to confront the suppression impact of osmotic stress and reawakened varied suppressed genes. SAL also induced the expression of stress-related genes, particularly *GmGOGAT, GmUBC2, ZmpsbA, ZmNAGK, ZmVPP1*, and *ZmSCE1d*. Overall, our data provided confirmation for the evidence that salvianolic acid can noticeably strengthen resistance to osmotic stress and therefore can be a potential candidate to be utilized in agriculture.

## Data Availability Statement

The original contributions presented in the study are included in the article/Supplementary Material, further inquiries can be directed to the corresponding authors.

## Author Contributions

EK conceived the project, designed the experiments, performed the experiments, analyzed the data, and wrote the manuscript. AA-S edited the manuscript. UR helped in data analysis. I-DK, S-MK, and I-JL provided the resources. All authors approved the final manuscript.

## Funding

This research was supported by the National Research Foundation of Korea (NRF) grant funded by the Korean government (MSIT) (No. 2022R1A2C1008993).

## Conflict of Interest

The authors declare that the research was conducted in the absence of any commercial or financial relationships that could be construed as a potential conflict of interest.

## Publisher's Note

All claims expressed in this article are solely those of the authors and do not necessarily represent those of their affiliated organizations, or those of the publisher, the editors and the reviewers. Any product that may be evaluated in this article, or claim that may be made by its manufacturer, is not guaranteed or endorsed by the publisher.
