# Supplementary material for: Salvianolic Acid Modulates Physiological Responses and Stress-Related Genes That Affect Osmotic Stress Tolerance in Glycine max and Zea mays
Source: Front Plant Sci. 2022 Jun 15;13:904037. doi: 10.3389/fpls.2022.904037 (PMC9240475; doi:10.3389/fpls.2022.904037)
Supplement: Supplementary file 1 [file Data_Sheet_1.docx]

**Supplementary materials**

**Salvianolic acid modulates physiological responses and stress-related genes to affect osmotic stress tolerance in Glycine max and *Zea mays***

Elham Ahmed Kazerooni^1*^, Abdullah Mohammed Al-Sadi^2^, Umer Rashid^3^, Il-Doo Kim^1^, Sang-Mo Kang^1^, In-Jung Lee^1*^

^1^Department of Applied Biosciences, Kyungpook National University, Daegu 41566, Republic of Korea

^2^Department of Plant Sciences, College of Agricultural and Marine Sciences, Sultan Qaboos University, PO Box 34, Al-Khod 123, Oman

^3^Institute of Nanoscience and Nanotechnology (ION2), Universiti Putra Malaysia, Serdang, Selangor, Malaysia

**^*^Corresponding authors**

Prof. In-Jung lee

Dr. Elham Ahmed Kazerooni

Crop Physiology Laboratory

Department of Applied Biosciences

Kyungpook National University

Daegu 41566, Republic of Korea

E-mail: [ijlee@knu.ac.kr](mailto:ijlee@knu.ac.kr)

E-mail: [elham.ghasemi.k@gmail.com](mailto:elham.ghasemi.k@gmail.com)

Tel: +82-53-950-5708(Office)

**
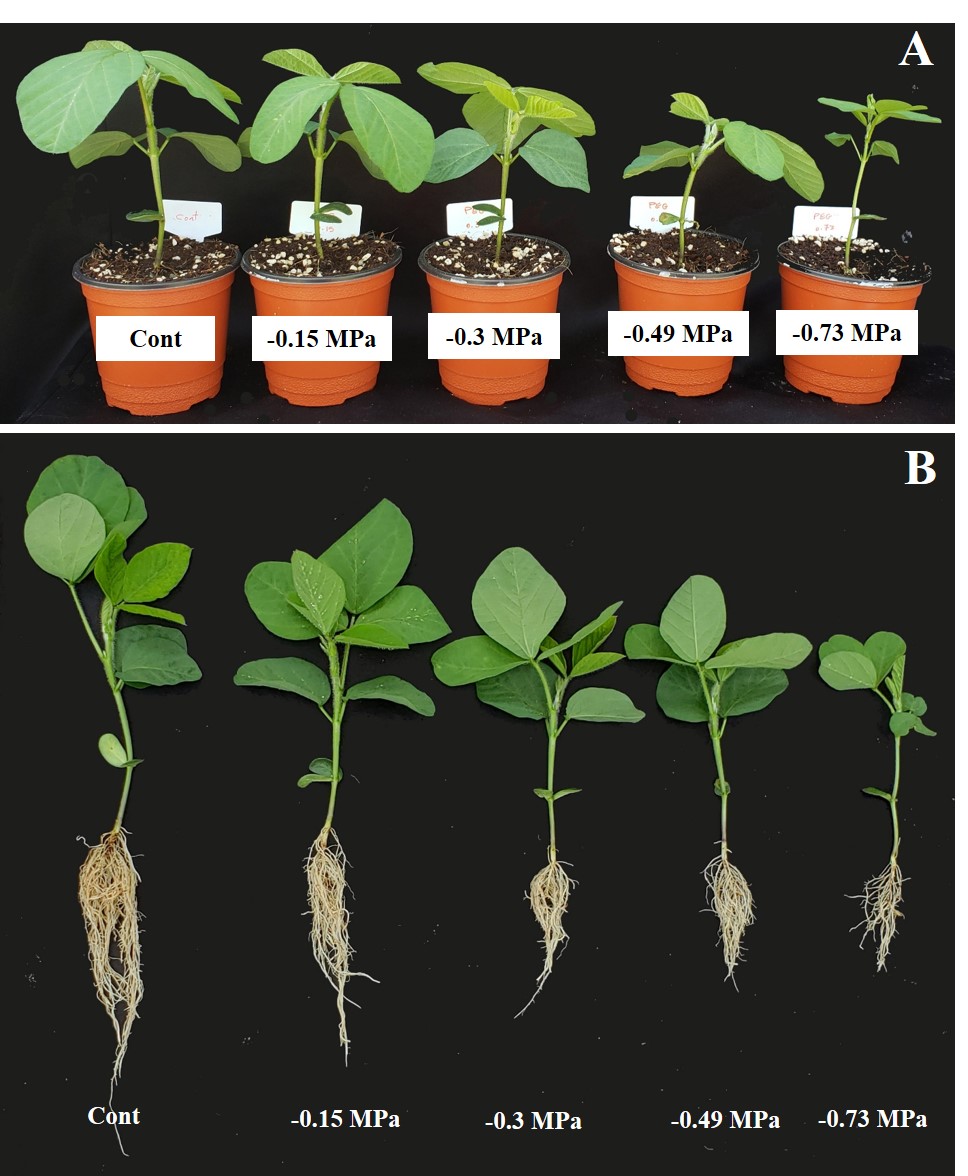
**

**Figure S1.** Effects of various PEG (Polyethylene glycol) concentrations on the growth of soybean seedlings following 8 days of treatment (8DAT; A and B). Treatments: Cont (control), PEG (-0.15 MPa), PEG (-0.3 MPa), PEG (-0.49 MPa), and PEG (-0.73 MPa).


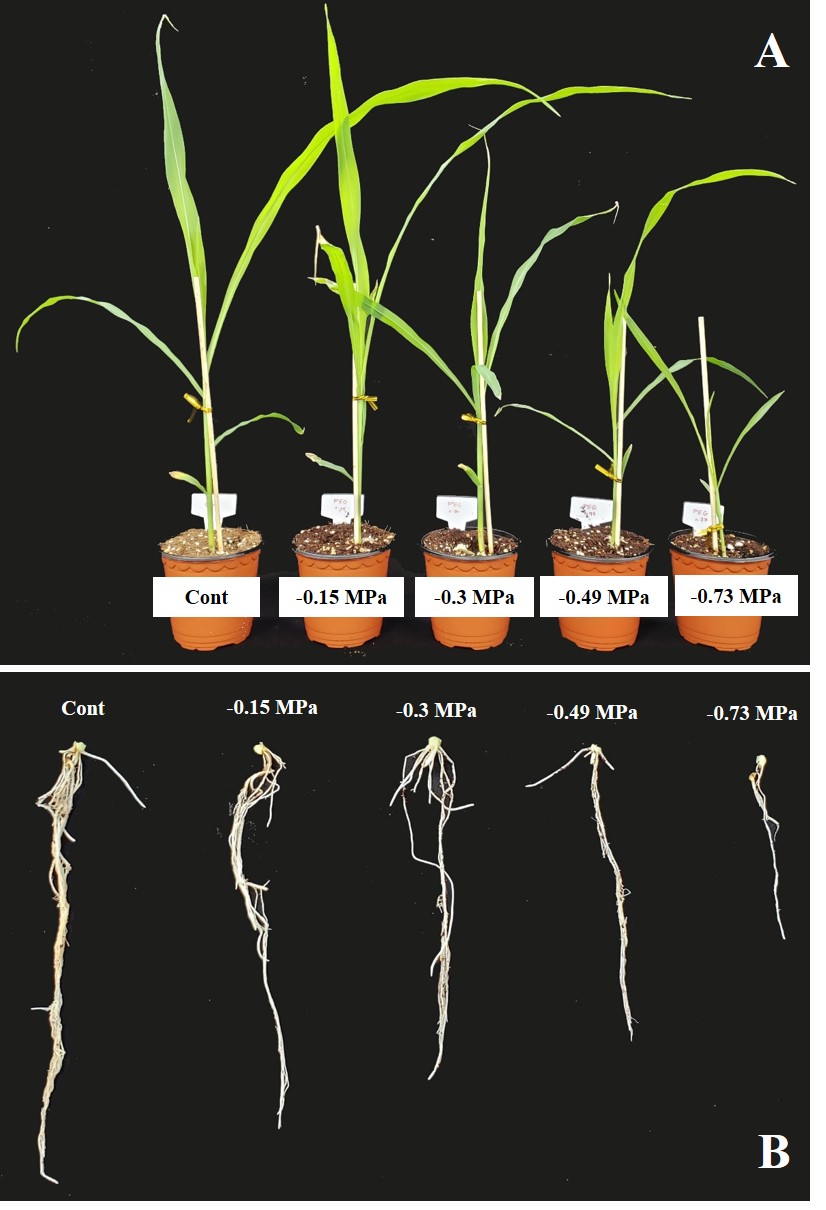


**Figure S2.** Effects of various PEG (Polyethylene glycol) concentrations on the growth of maize seedlings following 8 days of treatment (8DAT; A and B). Treatments: Cont (control), PEG (-0.15 MPa), PEG (-0.3 MPa), PEG (-0.49 MPa), and PEG (-0.73 MPa).


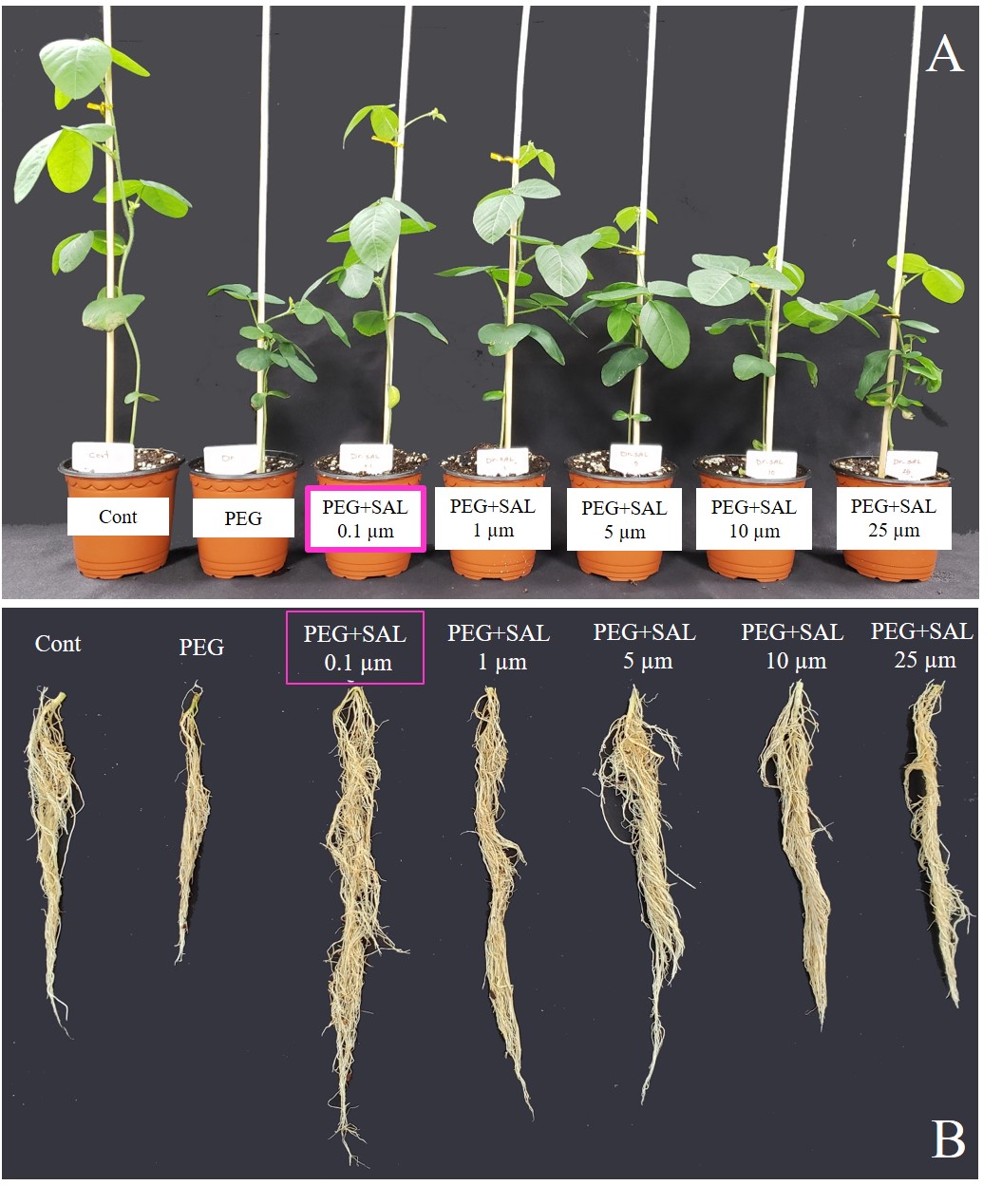


**Figure S3.** Effects of various salvianolic acid concentrations on the growth of stressed-soybean seedlings following 8 days of polyethelene glycol treatment (8DAT; A and B). Treatments: Cont (control), PEG (25% polyethelene glycol), SAL (0.1 μM salvianolic acid) + PEG (25% polyethelene glycol), SAL (1 μM salvianolic acid) + PEG (25% polyethelene glycol), SAL (5 μM salvianolic acid) + PEG (25% polyethelene glycol), SAL (10 μM salvianolic acid) + PEG (25% polyethelene glycol), and SAL (25 μM salvianolic acid) + PEG (25% polyethelene glycol).


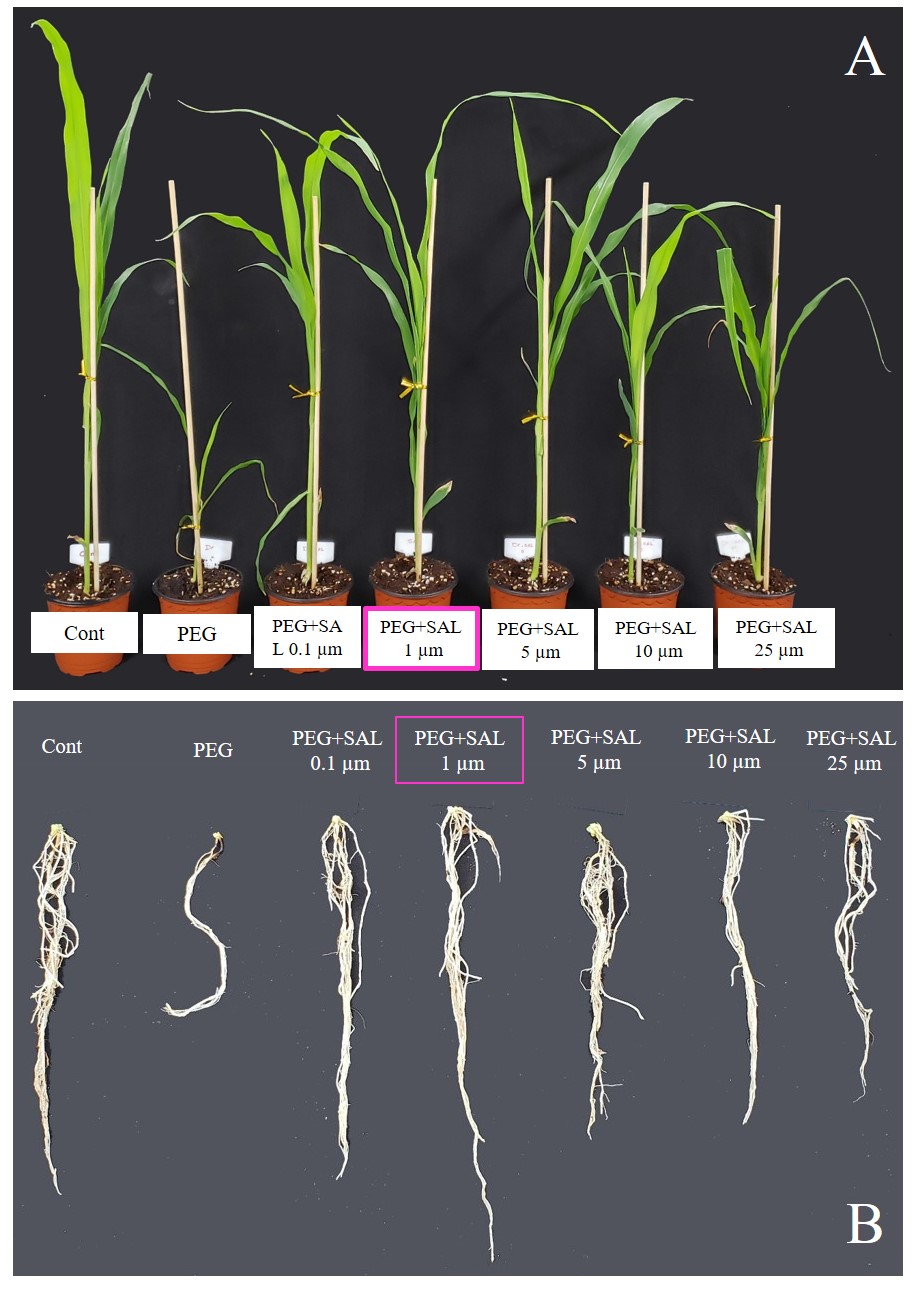


**Figure S4.** Effects of various salvianolic acid concentrations on the growth of stressed-maize seedlings following 8 days of polyethelene glycol treatment (8DAT; A and B). Treatments: Cont (control), PEG (25% polyethelene glycol), SAL (0.1 μM salvianolic acid) + PEG (25% polyethelene glycol), SAL (1 μM salvianolic acid) + PEG (25% polyethelene glycol), SAL (5 μM salvianolic acid) + PEG (25% polyethelene glycol), SAL (10 μM salvianolic acid) + PEG (25% polyethelene glycol), and SAL (25 μM salvianolic acid) + PEG (25% polyethelene glycol).

**Table S1.** Primers used for relative gene expression analysis.

| **Gene symbol** | **Primers (5′–3′) Forward/reverse** |
| --- | --- |
|  |  |
| GmUBC2 | 5′-CGGCGAATTCATGTCGACTCCTGCTAGGAA-3′ |
|  | 5′-GCCGAAGCTTTTAATCTGCTGTCCAACTCTGC-3′ |
| GmACS | 5′-CTCTTAACCTTCATTCTTGCTAACC-3′ |
|  | 5′-TTGCTTCTGCTTCTTTGTATGC-3′ |
| GmCKX | 5′-TTGGGTCACGATGGGAGGCA-3′ |
|  | 5′-TGGCCAGGGGCTAGAAGTGCT-3′ |
| GmMIPS2 | 5′-GTTGCTACCATCCTCAGCTACC-3′ |
|  | 5′-TCATTTTGGTCTGACCACTCT-3′ |
| GmGOGAT | 5′-ACACTCTCATCTTCTCTCTCTCTCTCTCTCG-3′ |
|  | 5′-CGTTGTGGAGGGAAGGGAATG-3′ |
| GmSOG1 | 5′-CTCTTCTTCGATACAATGGCTAGG-3′ |
|  | 5′-CGCATAACCTGTCCATCCAATCAA-3′ |
| GmActin | 5′-GCGTGATCTCACTGATGCCCTTAT-3′ |
|  | 5′-AGCCTTCGCAATCCACATCTGTTG-3′ |

**Table S2.** Primers used for relative gene expression analysis.

| **Gene symbol** | **Primers (5′–3′) Forward/reverse** |
| --- | --- |
|  |  |
| ZmNAGK | 5'-ATGGTCCTCACGAAACCCTA-3' |
|  | 5'-GCCAGTGATCATGGTGCC-3' |
| ZmpsbA | 5'-CAATTTTAGAGAGACGCGAAAGTAC-3' |
|  | 5'-GTAAAAATGC AATCCGATCG CC-3' |
| ZmVPP1 | 5'-TATACGTGTCAAAAGCTC-3' |
|  | 5'-CGGATAATTTCGTGTGGA-3' |
| ZmNAC48 | 5'-CCTCCTGGTGGGCGTGAAGAG-3' |
|  | 5'-CGAGGCGGTACTCGTGCATGAC-3' |
| ZmPIS | 5'-CTCACCAATTTAAGGGCAGAAC-3' |
|  | 5'-TACATTTGAAACCAGTGGCTCG-3' |
| ZmSCE1d | 5'-CAGGAACACGTCGCTTATATTCTGC-3 |
|  | 5'-TCTGCCTCTCAATTCTGAAC-3' |
| ZmActin | 5'-GGCAAGTCATCACGATTGG-3' |
|  | 5'-CAGCTTCCATTCCCACAAAC-3' |

**Table S3**. Effects of various PEG (Polyethylene glycol) concentrations on the growth of soybean seedlings. Data were calculated after 8 days of treatment (8DAT).

| **Treatment** | **Plant height** | **Root length** | **Stem diameter** | **Leaf length** | **Leaf width** | **Chl** | **Plant fresh weight** | **Plant dry weight** | **Root fresh weight** | **Root dry weight** | **No. leaf** |
| --- | --- | --- | --- | --- | --- | --- | --- | --- | --- | --- | --- |
| 8DAT | (cm) | (cm) | (cm) | (cm) | (cm) | (Spad) | (g) | (g) | (g) | (g) |  |
| Soybean |  |  |  |  |  |  |  |  |  |  |  |
| Cont | 15.66±0.33a | 21.16±0.60a | 0.33±0.0a | 8.18±0.0a | 6.38±0.03a | 37.96±1.47a | 5.17±0.38a | 0.54±0.01a | 2.12±0.04a | 0.15±0.0a | 13.66±0.33a |
| PEG (-0.15) | 11.33±0.33b | 14.50±0.50b | 0.30±0.0b | 7.13±0.01b | 4.19±0.04b | 28.66±0.63b | 4.76±0.24a | 0.52±0.01a | 1.25±0.04b | 0.08±0.0b | 11.33±0.33b |
| PEG (-0.30) | 9.33±0.33c | 9.40±0.05c | 0.24±0.0c | 5.79±0.10c | 3.71±0.07c | 25.46±0.75c | 3.45±0.10b | 0.45±0.01b | 0.71±0.0c | 0.04±0.0c | 9.33±0.33c |
| PEG (-0.49) | 8.33±0.33cd | 7.36±0.08d | 0.20±0.0d | 4.23±0.07d | 3.36±0.08d | 20.53±0.39d | 2.50±0.01c | 0.34±0.0c | 0.50±0.04d | 0.03±0.0c | 8.66±0.66c |
| PEG (-0.73) | 7.33±0.33d | 5.33±0.33e | 0.18±0.0e | 3.76±0.08e | 3.15±0.04e | 19.70±0.34d | 1.17±0.01d | 0.11±0.0d | 0.26±0.01e | 0.01±0.0d | 8.33±0.33c |

Treatments: Cont (control), PEG (-0.15 MPa), PEG (-0.3 MPa), PEG (-0.49 MPa), and PEG (-0.73 MPa). Values show the means ± SE (n = 6) and significant differences at p < 0.05 (Tukey test). Data within the same column followed by different lowercase letters are significantly different.

**Table S4**. Effects of various PEG (Polyethylene glycol) concentrations on the growth of maize seedlings. Data were calculated after 8 days of treatment (8DAT).

| **Treatment** | **Plant height** | **Root length** | **Stem diameter** | **Leaf length** | **Leaf width** | **Chl** | **Plant fresh weight** | **Plant dry weight** | **Root fresh weight** | **Root dry weight** | **No. leaf** |
| --- | --- | --- | --- | --- | --- | --- | --- | --- | --- | --- | --- |
| 8DAT | (cm) | (cm) | (cm) | (cm) | (cm) | (Spad) | (g) | (g) | (g) | (g) |  |
| Maize |  |  |  |  |  |  |  |  |  |  |  |
| Cont | 31.33±0.88a | 22.0±0.57a | 0.38±0.0a | 48.66±0.33a | 2.40±0.11a | 35.8±0.85a | 5.30±0.01a | 0.38±0.0a | 1.21±0.0a | 0.07±0.0a | 6.33±0.33a |
| PEG (-0.15) | 24.0±0.57b | 17.33±0.33b | 0.32±0.0b | 41.66±0.66b | 2.03±0.03b | 27.83±1.10b | 4.15±0.01b | 0.30±0.0b | 0.72±0.01b | 0.04±0.0b | 5.0±0.0b |
| PEG (-0.30) | 18.66±0.33c | 16.16±0.16b | 0.31±0.0c | 33.0±0.57c | 1.46±0.08c | 24.83±0.53bc | 3.03±0.03c | 0.22±0.01c | 0.65±0.01c | 0.02±0.0c | 4.0±0.0c |
| PEG (-0.49) | 15.66±0.33d | 14.33±0.33c | 0.24±0.0d | 29.66±0.88d | 1.13±0.06d | 23.46±0.26c | 2.09±0.10d | 0.17±0.0d | 0.42±0.01d | 0.01±0.0d | 3.66±0.33c |
| PEG (-0.73) | 13.0±0.57e | 11.0±0.57d | 0.16±0.0e | 19.66±0.66e | 0.76±0.03e | 17.46±2.59d | 0.61±0.02e | 0.04±0.0e | 0.08±0.0e | 0.0±0.0d | 3.0±0.0d |

Treatments: Cont (control), PEG (-0.15 MPa), PEG (-0.3 MPa), PEG (-0.49 MPa), and PEG (-0.73 MPa). Values show the means ± SE (n = 6) and significant differences at p < 0.05 (Tukey test). Data within the same column followed by different lowercase letters are significantly different.

**Table S5.** Effects of various salvianolic acid (SAL) concentrations on the growth of osmotic (25% PEG; -0.73 MPa) stressed soybean and maize seedlings. Data were calculated after 8 days of treatment (8DAT).

| **Treatment** | **Plant height** | **Root length** | **Stem diameter** | **Leaf length** | **Leaf width** | **Chl** | **Plant fresh weight** | **Plant dry weight** | **Root fresh weight** | **Root dry weight** | **No. leaf** |
| --- | --- | --- | --- | --- | --- | --- | --- | --- | --- | --- | --- |
| 8DAT | (cm) | (cm) | (cm) | (cm) | (cm) | (Spad) | (g) | (g) | (g) | (g) |  |
| Soybean | | | | | | | | | | | |
| Cont | 35.33±0.33a | 17.33±0.16c | 0.42±0.01a | 6.13±0.03a | 4.36±0.03a | 32.70±0.35ab | 3.66±0.03a | 0.37±0.0a | 2.39±0.03a | 0.13±0.0a | 17.0±0.0a |
| PEG | 13.33±0.33g | 13.0±0.57f | 0.18±0.0f | 3.03±0.03g | 2.23±0.06e | 22.56±1.63e | 1.65±0.0f | 0.20±0.0f | 1.23±0.0f | 0.05±0.0d | 11.33±0.33e |
| PEG+SAL (0.1 µm) | 32.66±0.33b | 22.16±0.16a | 0.40±0.0b | 5.93±0.06b | 4.26±0.03a | 33.60±0.30a | 3.63±0.03a | 0.36±0.0a | 2.35±0.0ab | 0.12±0.0a | 15.33±0.33b |
| PEG+SAL (1 µm) | 22.83±0.16c | 20.10±0.20b | 0.39±0.0b | 5.66±0.03c | 4.06±0.03b | 32.56±0.51ab | 3.46±0.03b | 0.33±0.0b | 2.28±0.03bc | 0.10±0.0b | 14.0±0.0c |
| PEG+SAL (5 µm) | 20.83±0.44d | 19.3±0.15b | 0.36±0.0c | 5.10±0.10d | 4.03±0.03b | 31.0±0.57bc | 3.21±0.01c | 0.31±0.0c | 2.24±0.02c | 0.09±0.0b | 12.66±0.33d |
| PEG+SAL (10 µm) | 18.16±0.16e | 16.16±0.16d | 0.34±0.0d | 4.66±0.06e | 3.26±0.03c | 29.33±0.33cd | 3.03±0.03d | 0.28±0.0d | 2.14±0.03d | 0.08±0.0c | 11.66±0.33e |
| PEG+SAL (25 µm) | 16.66±0.33f | 15.0±0.28e | 0.31±0.0e | 4.30±0.05f | 2.90±0.05d | 27.33±0.33d | 2.76±0.06e | 0.25±0.0e | 1.52±0.01e | 0.06±0.0d | 10.33±0.33f |
| Maize | | | | | | | | | | | |
| Cont | 70.33±0.33a | 21.66±0.33bc | 0.51±0.0a | 55.33±0.33a | 1.97±0.0a | 32.80±0.11a | 10.37±0.0a | 2.09±0.0a | 1.36±0.0b | 0.08±0.0a | 6.66±0.33a |
| PEG | 21.33±0.88f | 13.0±0.57f | 0.22±0.0g | 13.66±0.33f | 0.78±0.0f | 20.83±0.88e | 0.97±0.0e | 0.07±0.0g | 0.32±0.0g | 0.02±0.0d | 4.0±0.0d |
| PEG+SAL (0.1 µm) | 56.33±0.66c | 21.33±0.33c | 0.42±0.0d | 39.33±0.33d | 1.84±0.0c | 30.73±0.14b | 6.22±0.01d | 0.97±0.0d | 1.26±0.0d | 0.06±0.0b | 6.0±0.0b |
| PEG+SAL (1 µm) | 69.33±0.66a | 28.0±0.57a | 0.48±0.0b | 54.66±0.33a | 1.97±0.0a | 32.80±0.80a | 9.36±0.37b | 1.92±0.02b | 1.66±0.0a | 0.08±0.0a | 7.0±0.0a |
| PEG+SAL (5 µm) | 58.66±0.33b | 22.66±0.33b | 0.43±0.0c | 49.66±0.33b | 1.93±0.0b | 27.30±0.35c | 7.94±0.0c | 1.61±0.0c | 1.32±0.0c | 0.07±0.0ab | 6.0±0.0b |
| PEG+SAL (10 µm) | 53.66±0.33d | 18.33±0.33d | 0.42±0.0e | 42.66±0.33c | 1.46±0.0d | 25.76±0.39c | 6.12±0.01d | 0.88±0.0e | 0.95±0.0e | 0.04±0.0c | 6.0±0.0b |
| PEG+SAL (25 µm) | 37.66±1.45e | 16.33±0.33e | 0.41±0.0f | 36.33±0.33e | 1.36±0.0e | 24.10±0.58d | 6.06±0.0d | 0.84±0.02f | 0.69±0.01f | 0.03±0.0cd | 5.33±0.33c |

Values show the means ± SE (n = 6) and significant differences at p < 0.05 (Tukey test). Data within the same column followed by different lowercase letters are significantly different.
